# Pensions without Penalties

**Published:** 1892-04-23

**Authors:** 


### Passing Topics.

### PENSIONS WITHOUT PENALTIES.

The proposal to give their liberty to the old gentlemen pensioners of the Royal Medical Benevolent College at Epsom must commend itself to everyone who holds misfortune in wholesome respect. Men of the type who inhabit the "furnished rooms" which represent a not inconsiderable portion of the gift the profession makes to its aged and needy members, are among the last to be insensible to those pleasures of a real home, and these are but poorly replaced by institutional care and comfort, however lavishly bestowed.

It is, we hope, a sign of a growing sympathy for respectable poverty that the disposition to brand it is becoming manifestly less common. As long as there are people in the world who are merely "successful," poverty will be associated, no doubt, in some measure with disgrace. The parvenu can no more rid himself of his spots than the leopard, but taken altogether the better part of humanity is, we think, distinctly more tolerant than it was, not only of honest labour, but of the straits to which a man who has laboured well is often reduced by no fault of his own. Even workhouse folk occasionally have their sensibilities allowed for, and it was at once a graceful act and a wise one for the guardians of one of the Metropolitan Unions to allow, as we recently read they have allowed, certain classes of their inmates to wear private clothes when absent on leave.

In the case of the Union we are, of course, prepared for the venerable argument that life in it must not be made too enticing, but when we come to a private charity engaged in the relief of unsuccessful members of a liberal profession, it is preposterous to suppose that to render its benefits more acceptable would cause duties and opportunities to be alike thrown to the winds in a reckless reliance upon philanthropic favours. The medical profession has a distinct claim upon our sympathies, and it is one which we fear is not too generally recognised. We have it on the authority of Dr. Wakley that all of the poor gentlemen concerned with whom he has conversed are anxious to return to live among their friends. No wonder; there is a sense of desolation and solitude with an unsatisfied craving of the heart, which will make themselves felt under such circumstances as are inseparable from the life of an asylum.

It is a wholly unnatural proceeding, and if not justified by necessity, barbarous, to separate the victims of misfortune from the world and the society which might sweeten their existence and imprison them with their own and like sorrows. There is room for reform in this direction in respect of not a few of our charities. In the present case a double advantage will be gained by setting the pensioners free. The space they occupy will become available for the school and the utility of this most important institution will be greatly extended. Of course it is a question of funds, but these we may hope will be forthcoming when the need becomes known, and the pensioners will thus cease to be penalised in residential conditions which detract greatly from the kindly and considerate character of the assistance afforded them.

Agricola.

